# Efficacy, Safety, and Pharmacokinetics by Body Mass Index Category in Phase 3/3b Long-Acting Cabotegravir Plus Rilpivirine Trials

**DOI:** 10.1093/infdis/jiad580

**Published:** 2023-12-22

**Authors:** Emilie R Elliot, Joseph W Polli, Parul Patel, Louise Garside, Richard Grove, Vincent Barnett, Jeremy Roberts, Sri Byrapuneni, Herta Crauwels, Susan L Ford, Rodica Van Solingen-Ristea, Eileen Birmingham, Ronald D’Amico, Bryan Baugh, Jean van Wyk

**Affiliations:** ViiV Healthcare, Durham, North Carolina, USA; ViiV Healthcare, Durham, North Carolina, USA; ViiV Healthcare, Durham, North Carolina, USA; GSK, London, United Kingdom; GSK, Uxbridge, United Kingdom; GSK, Durham, North Carolina, USA; GSK, Mississauga, Ontario, Canada; Parexel International, Research Triangle Park, North Carolina, USA; Janssen Research and Development, Beerse, Belgium; GSK, Durham, North Carolina, USA; Janssen Research and Development, Beerse, Belgium; Janssen Research and Development, Raritan, New Jersey, USA; ViiV Healthcare, Durham, North Carolina, USA; Janssen Research and Development, Raritan, New Jersey, USA; ViiV Healthcare, Brentford, United Kingdom

**Keywords:** BMI, cabotegravir, HIV-1, long-acting, rilpivirine

## Abstract

**Background:**

Cabotegravir plus rilpivirine (CAB + RPV) is a guideline-recommended long-acting (LA) injectable regimen for the maintenance of human immunodeficiency virus-1 (HIV-1) virologic suppression. This post hoc analysis summarizes CAB + RPV LA results by baseline body mass index (BMI) category among phase 3/3b trial participants.

**Methods:**

Data from CAB + RPV-naive participants receiving every 4 or 8 week dosing in FLAIR, ATLAS, and ATLAS-2M were pooled through week 48. Data beyond week 48 were summarized by study (FLAIR through week 96 and ATLAS-2M through week 152). HIV-1 RNA <50 and ≥50 copies/mL, confirmed virologic failure (CVF; 2 consecutive HIV-1 RNA ≥200 copies/mL), safety and tolerability, and plasma CAB and RPV trough concentrations were evaluated by baseline BMI (<30 kg/m^2^, lower; ≥30 kg/m^2^, higher).

**Results:**

Among 1245 CAB + RPV LA participants, 213 (17%) had a baseline BMI ≥30 kg/m^2^. At week 48, 92% versus 93% of participants with lower versus higher BMI had HIV-1 RNA <50 copies/mL, respectively. Including data beyond week 48, 18 participants had CVF; those in the higher BMI group (n = 8) all had at least 1 other baseline factor associated with CVF (archived RPV resistance-associated mutations or HIV-1 subtype A6/A1). Safety and pharmacokinetic profiles were comparable between BMI categories.

**Conclusions:**

CAB + RPV LA was efficacious and well tolerated, regardless of baseline BMI category.

**Clinical Trials Registration:**

NCT02938520, NCT02951052, and NCT03299049.

## Teaser

CAB + RPV LA is effective in the maintenance of HIV-1 virologic suppression in adults regardless of BMI category, with longer-length needles (≥2 inches) recommend for those with BMI ≥30 kg/m^2^ to accommodate individual body habitus and ensure appropriate administration.

Antiretroviral therapy (ART) consists of a combination of ≥2 agents from at least 2 drug classes [1–3]. Cabotegravir (CAB), an integrase strand transfer inhibitor (INSTI), plus rilpivirine (RPV), a nonnucleoside reverse transcriptase inhibitor (NNRTI), is the first complete long-acting (LA) ART regimen, administered monthly (Q1M) or every 2 months (Q2M) via intramuscular injection, recommended by treatment guidelines for the maintenance of human immunodeficiency virus-1 (HIV-1) virologic suppression [1–3]. LA injectable regimens have the potential to address some of the psychosocial stressors associated with daily oral ART that are experienced by some people with HIV (PWH) [4]. Treatment guidelines recognize this potential, including the US Department of Health and Human Services guidelines, which state that PWH who are interested in nonoral ART options due to concerns surrounding privacy, stigma, or convenience have demonstrated greater satisfaction with CAB + RPV LA than continued oral therapy [1].

CAB + RPV LA has demonstrated favorable efficacy and safety with a low rate (approximately 1%) of confirmed virologic failure (CVF) in phase 3/3b clinical trials (FLAIR, ATLAS, and ATLAS-2M) [5–12]. Additionally, numerically lower CVF rates (0%–0.5%) have been reported in the phase 3/3b CARISEL (Q2M) and CUSTOMIZE (Q1M) implementation studies, the Q2M CARLOS real-world study, and the Q2M SOLAR head-to-head trial versus bictegravir/emtricitabine/tenofovir alafenamide [13–16]. While few (0%–1%) participants experienced CVF in these studies, consideration of baseline factors can guide clinicians in patient identification and help minimize CVF risk. The presence of ≥2 baseline factors (of the following 3: preexisting RPV resistance-associated mutations [RAMs], HIV-1 subtype A6/A1, and/or body mass index [BMI] ≥30 kg/m^2^) was associated with an increased risk of CVF on LA therapy in a post hoc analysis of the FLAIR, ATLAS, and ATLAS-2M studies up to 152 weeks [17, 18]. In this analysis, the CVF rate was very low for participants with no or 1 baseline factor; more specifically, participants with BMI ≥30 kg/m^2^ as their only factor had a CVF rate of 0.5%—similar to those with no factors (0.4%) [17].

The global prevalence of obesity (BMI ≥30 kg/m^2^) has been rising and is associated with many comorbidities [19]. Obesity has the potential to affect drug absorption of medications administered by the intramuscular route, as well as the incidence of inadvertent administration into subcutaneous tissues due to inadequate needle length [19, 20].

To examine the potential impact of obesity on treatment outcomes for those receiving CAB + RPV LA, we conducted a post hoc analysis examining the efficacy, safety, and pharmacokinetics (PK) of CAB + RPV LA using pooled data through week 48 from participants in the FLAIR, ATLAS, and ATLAS-2M studies, stratified by baseline BMI category (<30 kg/m^2^, lower BMI group; ≥30 kg/m^2^, higher BMI group). Data beyond week 48 for FLAIR (week 96) and ATLAS-2M (week 152) are also described.

## METHODS

### Study Population

Data from participants without prior exposure to CAB + RPV who received CAB + RPV LA every 4 weeks (Q4W) or every 8 weeks (Q8W) dosing within the phase 3/3b FLAIR (NCT02938520), ATLAS (NCT02951052), and ATLAS-2M (NCT03299049) trials through week 48 were pooled in a post hoc analysis. Additionally, data beyond week 48 were summarized separately for FLAIR and ATLAS-2M participants (data beyond week 48 for the ATLAS study were not summarized as most participants transitioned to ATLAS-2M after week 48 during the extension phase of ATLAS).

FLAIR, ATLAS, and ATLAS-2M are randomized, multicenter, parallel-group, open-label, noninferiority phase 3/3b studies evaluating CAB + RPV LA dosed Q4W versus continuing daily oral therapy (FLAIR and ATLAS), or CAB + RPV LA dosed Q8W versus Q4W (ATLAS-2M). The full study designs and eligibility criteria have been published previously [6, 7, 9]. All studies included a screening phase, a maintenance phase, and an extension phase. Enrolled participants were 18 years of age or older and virologically suppressed with a plasma HIV-1 RNA <50 copies/mL prior to randomization without evidence of any major INSTI or NNRTI RAMs (except K103N). Baseline characteristics were generally similar across the 3 studies and across arms [6, 7, 9].

FLAIR participants were ART naive at study entry and underwent an induction phase with daily oral dolutegravir/abacavir/lamivudine for 20 weeks (participants who were HLA-B*5701-positive received a non-abacavir regimen) to achieve virologic suppression, while ATLAS and ATLAS-2M participants were virologically suppressed (HIV-1 RNA <50 copies/mL) on their current oral regimen at study entry. Most participants who received CAB + RPV LA in the ATLAS study rolled over to the ATLAS-2M study after week 48 (n = 391). ATLAS-2M data from participants who had rolled over from ATLAS with prior exposure to CAB + RPV were excluded to align duration of exposure and PK parameters in the 48-week pooled study population. Beyond week 48, data were summarized separately for FLAIR (week 96) and ATLAS-2M (week 152) as different time points were being evaluated; for ATLAS-2M, all participants (including those who entered the study with prior CAB + RPV exposure) were included in the summary. ATLAS data beyond week 48 were not summarized as most participants had transitioned to ATLAS-2M after week 48 [7, 10].

All 3 studies were conducted in accordance with the Declaration of Helsinki [21]. All participants provided written informed consent, and the study protocols, any amendments, informed consent, and other information that required preapproval were reviewed and approved by a national, regional, or investigational center ethics committee or institutional review board.

### End Points and Assessment

End points assessed by baseline BMI category were the proportion of participants with a plasma HIV-1 RNA <50 copies/mL and ≥50 copies/mL by the Food and Drug Administration (FDA) Snapshot algorithm (weeks 48, 96, and 152), the incidence of CVF (2 consecutive measurements of plasma HIV-1 RNA ≥200 copies/mL; baseline through week 152), adverse events (AEs; baseline through week 152) including injection site reactions (ISRs; week 4 through week 152), and CAB and RPV plasma trough concentrations (C_trough_; week 4 through week 96; including analysis of CAB concentrations by needle length for participants with a BMI ≥30 kg/m^2^, week 4 through week 48). Analysis of RPV concentration by needle length was not conducted as previous analyses have indicated that BMI has no impact on RPV LA absorption [22, 23]. PK plasma samples were analyzed for CAB and RPV concentrations using liquid chromatography with tandem mass spectrometry methods.

### Statistical Analysis

Participants were stratified by baseline BMI category (<30 kg/m^2^, lower BMI group; ≥30 kg/m^2^, higher BMI group) and CAB + RPV LA dosing regimen (Q8W and Q4W). All data presented in this post hoc analysis are descriptive.

## RESULTS

### Participants

In total, 1245 randomized participants (Q8W, n = 327; Q4W, n = 918) with a median baseline BMI of 25.1 kg/m^2^ (range, 15.30–54.02 kg/m^2^) were included in the week 48 analysis; 1032 (83%) had a baseline BMI <30 kg/m^2^ and 213 (17%) had a baseline BMI ≥30 kg/m^2^. Overall, 22% (n = 47 of 213) of participants in the higher BMI group had a baseline BMI of 35–<40 kg/m^2^, and 12% (n = 25 of 213) had a baseline BMI of ≥40 kg/m^2^. Median age was comparable across BMI categories and dosing regimens; however, there was a lower proportion of female (sex at birth) participants and black or African American participants in the lower BMI group versus the higher BMI group (Table 1). For data beyond week 48, 283 participants (86%, n = 243 of 283, in the lower BMI group; 14%, n = 40 of 283, in the higher BMI group) from FLAIR and 1045 participants (80%, n = 834 of 1045, in the lower BMI group; 20%, n = 211 of 1045, in the higher BMI group) from ATLAS-2M were included in the analysis.

**Table 1.** Baseline Characteristics for Pooled CAB + RPV LA Participants Across the ATLAS, FLAIR, and ATLAS-2M Clinical Trials Through Week 48

ITT-E Population	Pooled CAB + RPV LA Participants Across FLAIR, ATLAS, and ATLAS-2M			
	BMI <30 kg/m^2^ (n = 1032)		BMI ≥30 kg/m^2^ (n = 213)	
	Q8W (n = 268)	Q4W (n = 764)	Q8W (n = 59)	Q4W (n = 154)
Age, y, median (range)	41 (20–83)	38 (19–68)	43 (23–71)	41 (23–74)
≥50 y, n (%)	73 (27)	148 (19)	16 (27)	37 (24)
Female sex at birth, n (%)	48 (18)	172 (23)	25 (42)	65 (42)
Race, n (%)				
White	201 (75)	591 (77)	37 (63)	95 (62)
Black or African American	37 (14)	103 (13)	20 (34)	51 (33)
Asian	17 (6)	44 (6)	0	2 (1)
Other races	13 (5)	26 (3)	2 (3)	6 (4)
Hispanic or Latinx ethnicity, n (%)	43 (16)	89 (12)	11 (19)	16 (10)
Weight, kg, median (range)	74.0 (49.0–109.2)	73.2 (41.2–108.4)	98.0 (76.0–136.9)	99.9 (70.9–139.4)
BMI, kg/m^2^, median (range)	24.4 (17.8–30.0)	24.0 (15.3–29.9)	32.5 (30.1–46.0)	33.2 (30.0–54.0)
30 to <40, n (%)	NA	NA	49 (83)	139 (90)
≥40, n (%)	NA	NA	10 (17)	15 (10)

Abbreviations: BMI, body mass index; CAB, cabotegravir; ITT-E, intention-to-treat exposed; LA, long-acting; NA, not applicable; Q4W, every 4 weeks; Q8W, every 8 weeks; RPV, rilpivirine.

### Data Through Week 48

#### Efficacy

Viral suppression was high and comparable across BMI categories at week 48 (Table 2). Within the lower BMI group, 94% and 93% of participants in the Q8W and Q4W dosing regimen had HIV-1 RNA <50 copies/mL at week 48, respectively. In the higher BMI group, 92% of participants across both dosing regimens had HIV-1 RNA <50 copies/mL at week 48. Overall, 10 (1%) versus 11 (5%) participants had HIV-1 RNA ≥50 copies/mL in the lower and higher BMI groups, respectively. A higher proportion of participants had no virologic data at week 48 in the lower BMI group versus the higher BMI group, driven by a higher number of discontinuations related to AEs and other reasons.

**Table 2.** Summary of Pooled Study Outcomes at Week 48: Snapshot Analysis (ITT-E Population)

Parameter	Pooled CAB + RPV LA Participants Across FLAIR, ATLAS, and ATLAS-2M			
	BMI <30 kg/m^2^ (n = 1032)		BMI ≥30 kg/m^2^ (n = 213)	
	Q8W (n = 268)	Q4W (n = 764)	Q8W (n = 59)	Q4W (n = 154)
HIV-1 RNA <50 copies/mL	252 (94.0)	708 (92.7)	54 (91.5)	142 (92.2)
HIV-1 RNA ≥50 copies/mL	1 (0.4)	9 (1.2)	4 (6.8)	7 (4.5)
Data in window not below threshold	1 (0.4)	1 (0.1)	0	4 (2.6)
Discontinued for lack of efficacy	0	6 (0.8)	4 (6.8)	3 (1.9)
Discontinued for other reason while not below threshold	0	2 (0.3)	0	0
No virologic data in week 48 window	15 (5.6)	47 (6.2)	1 (1.7)	5 (3.2)
Discontinued due to AE or death	6 (2.2)	29 (3.8)	0	1 (0.6)
Discontinued for other reasons	9 (3.4)	18 (2.4)	1 (1.7)	4 (2.6)

Data are No. (%).

Abbreviations: AE, adverse event; BMI, body mass index; CAB, cabotegravir; ITT-E, intention-to-treat exposed; LA, long-acting; Q4W, every 4 weeks; Q8W, every 8 weeks; RPV, rilpivirine.

#### Confirmed Virologic Failure

Through 48 weeks of CAB + RPV LA therapy, 1% (n = 15 of 1246) of participants met the CVF criterion (Supplementary Table 1). Of these participants, 1 had oral CAB + RPV dosing interrupted due to a false-positive pregnancy test and, upon reinitiating oral therapy, had suspected virologic failure that was confirmed; this participant did not receive a CAB + RPV LA injection. Of the 14 participants who met the CVF criterion and received injections, 6 (<1%) and 8 (4%) occurred in the lower and higher BMI groups, respectively. Within the higher BMI group, all 8 participants with CVF had at least 1 other baseline factor previously associated with increased risk of CVF: RPV RAMs (n = 3), HIV-1 subtype A6/A1 (n = 4), or both (n = 1). Among the 153 participants with BMI ≥30 kg/m^2^ as their only baseline factor, none met the CVF criterion. Within the lower BMI group, 3 participants had no factors, 2 had HIV-1 subtype A6/A1, and 1 had both HIV-1 subtype A6/A1 and RPV RAMs.

#### Safety

##### AEs Excluding ISRs

The frequency of AEs was broadly comparable across BMI categories and dosing regimens, with 69%–86% of participants reporting an AE. Drug-related AEs were reported in 14%–29% of participants (Table 3). Drug-related grade ≥3 AEs were uncommon, occurring in 0%–2% of participants across both BMI categories and dosing regimens. Overall, 3% (n = 32 of 1032) and <1% (n = 1 of 213) of participants in the lower and higher BMI groups experienced AEs leading to withdrawal, respectively. Serious drug-related AEs occurred in 2 participants (<1%, both of whom were in the lower BMI group): right knee monoarthritis (n = 1, FLAIR) and hypersensitivity due to suspected (partial) intravenous administration of RPV (n = 1, ATLAS-2M). No fatal AEs occurred.

**Table 3.** Summary of AEs Through Week 48 (Excluding ISRs)

Parameter	Pooled CAB + RPV LA Participants Across FLAIR, ATLAS, and ATLAS-2M			
	BMI <30 kg/m^2^		BMI ≥30 kg/m^2^	
	Q8W (n = 268)	Q4W (n = 764)	Q8W (n = 59)	Q4W (n = 154)
Any AE	211 (79)	656 (86)	41 (69)	127 (82)
Any grade ≥3 AE	14 (5)	55 (7)	1 (2)	8 (5)
Any drug-related AE	66 (25)	218 (29)	8 (14)	43 (28)
Any grade ≥3 drug-related AE	2 (<1)	12 (2)	0	0
AE leading to withdrawal	6 (2)	26 (3)	0	1 (<1)
Any SAE^a^	13 (5)	30 (4)	2 (3)	5 (3)
Drug related	0	2 (<1)	0	0

Data are No. (%).

^a^None were fatal.

Abbreviations: AE, adverse event; BMI, body mass index; CAB, cabotegravir; ISR, injection site reaction; LA, long-acting; Q4W, every 4 weeks; Q8W, every 8 weeks; RPV, rilpivirine; SAE, serious adverse event.

##### ISR-specific AEs

**Figure 1.**
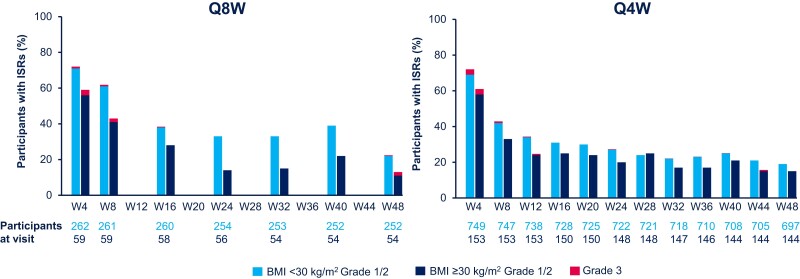
Summary of ISRs through week 48. AE grade is the maximum grade reported by the participant at each visit. Few ISRs were classified as grade 3 (approximately 1% of ISR events), consistent across both BMI categories and dosing regimens. There were no grade 4 or 5 ISR events. Abbreviations: AE, adverse event; BMI, body mass index; CAB, cabotegravir; ISR, injection site reaction; LA, long-acting; Q4W, every 4 weeks; Q8W, every 8 weeks; RPV, rilpivirine; W, week.

Through week 48, 24 399 and 4970 injections were administered resulting in 6655 and 1094 ISR events, in the lower and higher BMI groups, respectively. The ISR profiles were generally similar between dosing regimens, with slightly fewer pain events in the higher BMI group and fewer grade 3 ISRs in the lower BMI group (Supplementary Table 2). No grade 4 or 5 ISRs occurred. Overall, 3% (n = 34 of 1011) and 1% (n = 3 of 212) of participants in the lower and higher BMI groups had ISRs leading to withdrawal, respectively. Most ISRs were short lived (median, 3 days), with injection site pain being the most commonly reported (22% of all injections), regardless of BMI group or dosing regimen. ISR incidence decreased over time regardless of BMI group or dosing regimen, with a numerical trend toward fewer ISRs in the higher BMI group overall (Figure 1).

#### Pharmacokinetics

**Figure 2.**
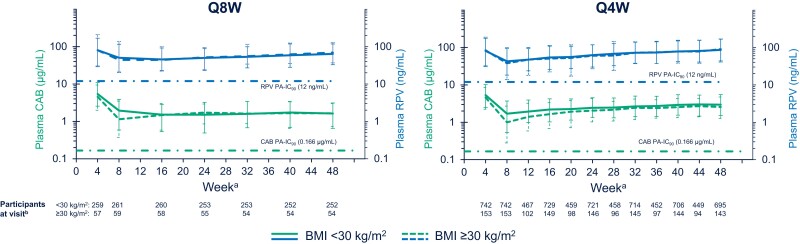
Median (5th and 95th percentile) plasma CAB and RPV troughs through week 48. Data beyond week 48 are not shown, as ATLAS-2M did not assess PK after week 48 except for the single values at week 96 and FLAIR PK were not consistently assessed after week 48. Participant numbers for CAB administration are shown. RPV participant numbers were identical, with the following exceptions: Q8W, <30 kg/m^2^: week 16, n = 259; Q8W, ≥30 kg/m^2^: week 4, n = 58; week 8, n = 58; Q4W, <30 kg/m^2^: week 8, n = 743; week 16, n = 730; week 24, n = 718; week 32, n = 715; week 44, n = 450; week 48, n = 691; Q4W, ≥30 kg/m^2^: week 4, n = 152; week 12, n = 101; week 24, n = 147; week 40, n = 143; week 48, n = 144. Abbreviations: BMI, body mass index; CAB, cabotegravir; PA-IC_90_, protein-adjusted 90% inhibitory concentration; PK, pharmacokinetics; Q4W, every 4 weeks; Q8W, every 8 weeks; RPV, rilpivirine.

CAB and RPV C_troughs_ remained above the respective protein-adjusted 90% inhibitory concentrations for both drugs (0.166 µg/mL for CAB and 12 ng/mL for RPV) regardless of baseline BMI category throughout the dosing period (Figure 2).

##### Cabotegravir

**Figure 3.**
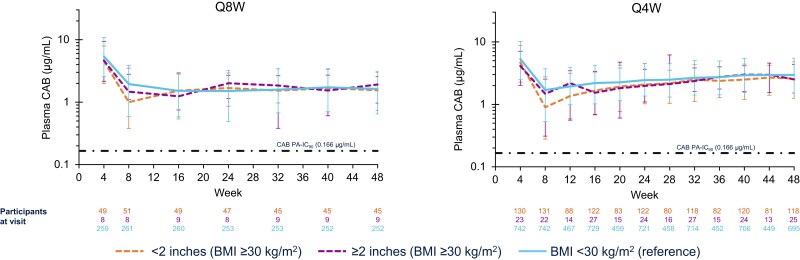
Median (5th and 95th percentile) plasma CAB C_trough_ through week 48 in participants with BMI ≥30 kg/m^2^ by needle length. The majority (78%, n = 3889 of 4970) of injections in participants with BMI ≥30 kg/m^2^ were administered with needles <1.6 inches (<4.06 cm) in length versus the recommended longer 2-inch (5.08 cm) needle due to issues with procurement. Abbreviations: BMI, body mass index; CAB, cabotegravir; C_trough_, trough concentration; PA-IC_90_, protein-adjusted 90% inhibitory concentration; Q4W, every 4 weeks; Q8W, every 8 weeks.

Median CAB C_troughs_ were initially slightly lower in participants in the higher BMI group versus those in the lower BMI group (Q8W, 1.14 vs 1.95 µg/mL; Q4W, 1.00 vs 1.70 µg/mL) at week 8, although by week 16 (1.48 vs 1.52 µg/mL) for Q8W dosing, and by week 28 (2.17 vs 2.48 µg/mL) for Q4W dosing, concentrations were similar between BMI groups through week 48. In the higher BMI group, the use of longer needle lengths (≥2 vs <2 inches [≥5.08 vs <5.08 cm]) resulted in higher CAB C_troughs_ early in treatment (Q8W: week 4, 4.65 vs 4.54 µg/mL, week 8, 1.47 vs 1.00 µg/mL, and week 16, 1.24 vs 1.53 µg/mL; Q4W: week 4, 4.11 vs 4.68 µg/mL, week 8, 1.47 vs 0.90 µg/mL, and week 12, 2.19 vs 1.36 µg/mL; Figure 3), although availability of longer needles was limited during the trials and they were used by a minority of participants in the higher BMI group (Q8W, n = 9 of 59, 15%; Q4W, n = 27 of 154, 18%). At week 48, median (5th and 95th percentile) CAB C_troughs_ were 1.63 µg/mL (0.65–3.12) in the lower BMI group and 1.65 µg/mL (0.74–3.02) in the higher BMI group for Q8W dosing, and 2.96 µg/mL (1.51–5.48) in the lower BMI group and 2.57 µg/mL (1.27–4.76) in the higher BMI group for Q4W dosing. Looking specifically at participants with a BMI ≥40 kg/m^2^, week 48 median (5th and 95th percentile) CAB C_troughs_ were 1.62 µg/mL (0.51–2.13) for Q8W dosing and 2.49 µg/mL (1.27–4.84) for Q4W dosing.

##### Rilpivirine

At week 48, median (5th and 95th percentile) RPV C_troughs_ were 63.85 ng/mL (32.9–118) in the lower BMI group and 69.45 ng/mL (36.8–128) in the higher BMI group for Q8W dosing and 86.40 ng/mL (43.8–167) in the lower BMI group and 90.50 ng/mL (40.8–170) in the higher BMI group for Q4W dosing. Looking specifically at participants with BMI ≥40 kg/m^2^, week 48 median (5th and 95th percentile) RPV C_troughs_ were 69.75 ng/mL (39.6–120) for Q8W dosing and 93.00 ng/mL (25.0–184) for Q4W dosing. Median RPV concentrations were similar between BMI categories throughout the dosing period.

### Data Beyond Week 48

#### Efficacy

High levels of virologic suppression were observed at week 96 (FLAIR) and week 152 (ATLAS-2M) and were comparable across BMI categories. At week 96 in FLAIR, 87% (n = 211 of 243) of participants in the lower BMI group and 85% (n = 34 of 40) in the higher BMI group maintained HIV-1 RNA <50 copies/mL (Supplementary Table 3). At week 152 in ATLAS-2M, virologic suppression was maintained in 87% (n = 725 of 834) and 85% (n = 180 of 211) of participants in the lower and higher BMI groups, respectively.

#### Confirmed Virologic Failure

Of the 18 participants with CVF (10 in the lower BMI group; 8 in the higher BMI group) through week 96 in FLAIR and week 152 in ATLAS-2M combined (excluding ATLAS; including those who entered ATLAS-2M with prior CAB + RPV exposure; Supplementary Table 4), 3 met the criterion after week 48; all 3 participants were in the lower BMI group. Of these 3 participants with CVF after week 48, 2 had at least 1 baseline factor associated with increased risk of CVF (1 participant had RPV RAMs, and the other participant had HIV-1 subtype A6/A1).

#### Safety

##### AEs Excluding ISRs

A summary of AEs (excluding ISRs) occurring between week 48 and week 96 in FLAIR and between week 48 and week 152 in ATLAS-2M is shown in Supplementary Table 5. In FLAIR, of those participants with no drug-related AEs in the week 0–48 period, 9% (n = 16 of 177) and 4% (n = 1 of 28) in the lower BMI and the higher BMI groups, respectively, had drug-related AEs (excluding ISRs) by week 96. In total, 4 (2%) participants in the lower BMI group had AEs leading to withdrawal after week 48; no AEs leading to withdrawal were reported for participants in the higher BMI group. There were no drug-related grade ≥3 or drug-related serious AEs (SAEs) reported beyond week 48 in FLAIR (Supplementary Table 5). In ATLAS-2M, of those participants with no drug-related AEs in the week 0–48 period, 10% (n = 63 of 636) and 7% (n = 12 of 175) in the lower and higher BMI groups, respectively, had drug-related AEs by week 152. Fourteen (2%) and 5 participants (2%) in the lower and higher BMI groups, respectively, withdrew due to AEs between week 48 and week 152. Since the week 48 analysis, 3 (<1%) participants in the lower BMI group had drug-related SAEs.

##### ISR AEs Over Time

The proportion of participants reporting ISRs generally decreased over time regardless of BMI group, with a numerical trend toward fewer ISRs in the higher BMI group (Supplementary Figure 1).

#### Pharmacokinetics

##### Cabotegravir

At week 96 in FLAIR, median (5th and 95th percentile) Q4W CAB C_trough_ were 2.73 µg/mL (1.44–5.25) and 2.62 µg/mL (1.27–4.67) in the lower and higher BMI groups, respectively, and were comparable with week 48 observations. At week 96 in ATLAS-2M, median CAB C_trough_ were similar to week 48 for both dosing regimens within the lower and higher BMI groups. Median (5th and 95th percentile) CAB C_trough_ at week 96 were 1.60 µg/mL (0.75–3.33) and 1.45 µg/mL (0.61–2.91) in the lower and higher BMI groups, respectively, for Q8W dosing and 2.81 µg/mL (1.48–5.21) and 2.58 µg/mL (1.06–4.48) in the lower and higher BMI groups, respectively, for Q4W dosing.

##### Rilpivirine

Median (5th and 95th percentile) RPV C_trough_ were broadly comparable between BMI groups at week 96 for FLAIR, with a slightly higher median concentration observed in the higher BMI group of 121 ng/mL (57.5–199) versus the lower BMI group of 108 ng/mL (58.9–221). An increase in RPV concentration was observed from week 48 to week 96 in ATLAS-2M (in both BMI categories and both dosing regimens), consistent with the half-life of RPV LA. Median (5th and 95th percentile) RPV C_trough_ at week 96 were 85.5 ng/mL (42.3–175) in the lower BMI group and 96.0 ng/mL (61.1–180) in the higher BMI group for Q8W dosing and 117 ng/mL (65.1–222) in the lower BMI group and 123 ng/mL (71.8–227) in the higher BMI group for Q4W dosing.

## DISCUSSION

The influence of BMI on efficacy and safety outcomes with ART has not been widely studied. Given that CAB + RPV LA is an injectable regimen, BMI is of particular interest, with previous PK analyses indicating that BMI values ≥30 kg/m^2^ are associated with initially slower CAB LA absorption [20, 24], but with no impact on RPV LA absorption [22]. Notably, baseline BMI (per unit increase) was associated with increased CVF risk in PWH receiving CAB + RPV LA in multivariable logistical regression analyses (adjusted incidence rate ratio, 1.09) [17, 18]. To evaluate the effect of BMI on virologic and safety outcomes with CAB + RPV LA, we pooled data through week 48 from 3 phase 3/3b trials, totaling 1245 PWH. Data beyond week 48 were also summarized to examine longer-term outcomes.

The efficacy results demonstrate that CAB + RPV LA Q4W and Q8W maintained high virologic suppression rates at week 48 (FDA Snapshot algorithm) regardless of baseline BMI category. High virologic suppression was also maintained beyond week 48, through week 96 in FLAIR, and through week 152 in ATLAS-2M. Rates of suppression in participants with a BMI <30 kg/m^2^ and ≥30 kg/m^2^ were consistent with overall virologic suppression rates observed in the primary analyses of the individual studies [6, 7, 9].

Through week 48, CVF was more frequent in participants with a BMI ≥30 kg/m^2^ (4% vs 1%, BMI <30 kg/m^2^) when combined with at least 1 other baseline factor; no participant with a BMI ≥30 kg/m^2^ as the only baseline factor met the CVF criterion through week 48. Additionally, none of the 3 participants with CVF after week 48 in FLAIR and ATLAS-2M had a BMI ≥30 kg/m^2^. These results are consistent with previous observations from expanded population multivariable analyses of studies with CAB + RPV, in which the incidence of CVF in participants with a BMI ≥30 kg/m^2^ as their sole baseline factor (0.5%) was similar to that of the overall population with no baseline factor through to week 152 (0.4%) [17].

The safety profiles were generally comparable between BMI categories through week 48 and up to week 152, with no new safety signals observed. Overall, the incidence of AEs leading to withdrawal was low for both BMI groups throughout week 152, consistent with the overall phase 3/3b population. Injections were well tolerated across both BMI categories, with most ISRs classified as mild to moderate in severity, the ISRs decreasing in incidence over time, and few ISRs leading to study withdrawal.

PK observations were consistent with population PK modeling data for both CAB and RPV [22, 23]. RPV concentrations were similar regardless of BMI category. Median CAB C_troughs_ tended to be lower in the first 16 weeks of therapy in participants with a baseline BMI ≥30 kg/m^2^ compared with the lower BMI group; this trend disappeared with drug accumulation after week 16. Importantly, higher median CAB C_trough_ were observed in the first 16 weeks when the longer-length needles (≥2 inches) were available and used for administration in participants with a BMI ≥30 kg/m^2^. This supports the product label that recommends longer-length needles to accommodate individual body habitus in participants with a BMI ≥30 kg/m^2^ and ensures appropriate administration into gluteal muscle [25]. When evaluating PK further in the higher BMI subgroup (BMI ≥40 kg/m^2^), C_troughs_ were also comparable.

This analysis had several limitations. Owing to the difference in dosing frequency, the Q4W arm received more frequent safety assessments than the Q8W arm, which may have increased the overall number of AEs reported in the Q4W arm for both BMI groups. There were more participants in the lower BMI group versus the higher BMI group; therefore, results should be interpreted with caution in view of the different group sizes. Furthermore, there were differing proportions of men versus women and race/ethnicities between the 2 BMI categories. The generalizability of the data is also limited for those with very high BMI values, given the small number of participants included who had a BMI ≥40 kg/m^2^ (<1%). Finally, no formal statistical analyses were performed; therefore, the data are purely descriptive.

## CONCLUSIONS

These data support the use of CAB + RPV LA dosed Q1M or Q2M as a complete regimen for the maintenance of HIV-1 virologic suppression in adults regardless of BMI category.

## Supplementary Data

Supplementary materials are available at *The Journal of Infectious Diseases* online (http://jid.oxfordjournals.org/). Supplementary materials consist of data provided by the author that are published to benefit the reader. The posted materials are not copyedited. The contents of all supplementary data are the sole responsibility of the authors. Questions or messages regarding errors should be addressed to the author.

## Supplementary Material

jiad580_Supplementary_Data

## Notes

***Acknowledgments.*** We thank everyone who has contributed to the FLAIR, ATLAS, and ATLAS-2M studies: all study participants and their families, and the clinical investigators and their staff. Professional medical writing and editorial assistance was provided by Poppy Mashilo of Scimentum (Nucleus Global), funded by ViiV Healthcare.

***Author contributions.*** All authors participated in the conceptualization and design of the studies, analysis and interpretation of the study data, and the drafting, review and approval of the manuscript.

***Data sharing.*** Data sharing requests will be considered by the management group upon written request to the corresponding author. Deidentified participant data or other prespecified data will be available subject to a written proposal and a signed data sharing agreement.

***Financial support*.** This work was supported by ViiV Healthcare. FLAIR, ATLAS, and ATLAS-2M were funded by ViiV Healthcare and Janssen Research and Development.
