# Supplementary material for: Efficacy, Safety, and Pharmacokinetics by Body Mass Index Category in Phase 3/3b Long-Acting Cabotegravir Plus Rilpivirine Trials
Source: J Infect Dis. 2023 Dec 22;230(1):e34–42. doi: 10.1093/infdis/jiad580 (PMC11272083; doi:10.1093/infdis/jiad580)
Supplement: jiad580_Supplementary_Data [file jiad580_supplementary_data.docx]

Figure S1. Summary of ISRs Through Week 96 (FLAIR [A]) and Week 152 (ATLAS-2M [B])


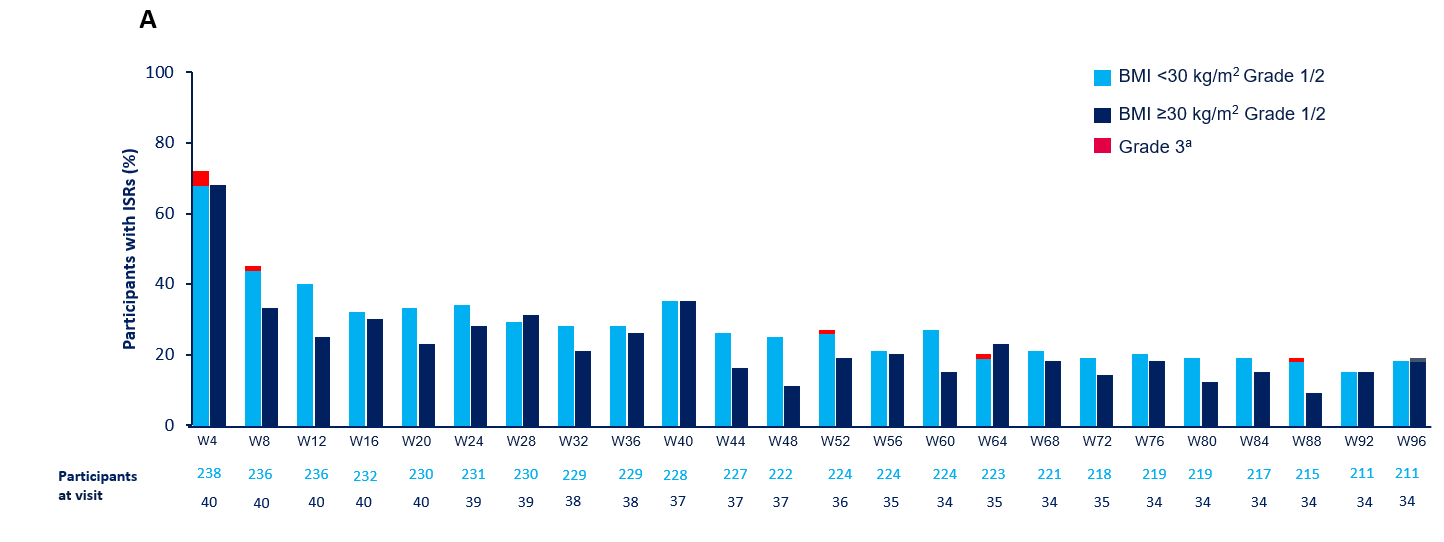


^a^AE grade is the maximum grade reported by the participant at each visit. Few ISRs were classified as Grade 3 (~1% of ISR events). There were no Grade 4 or 5 ISR events.

AE, adverse event; BMI, body mass index; ISR, injection site reaction; W, week.


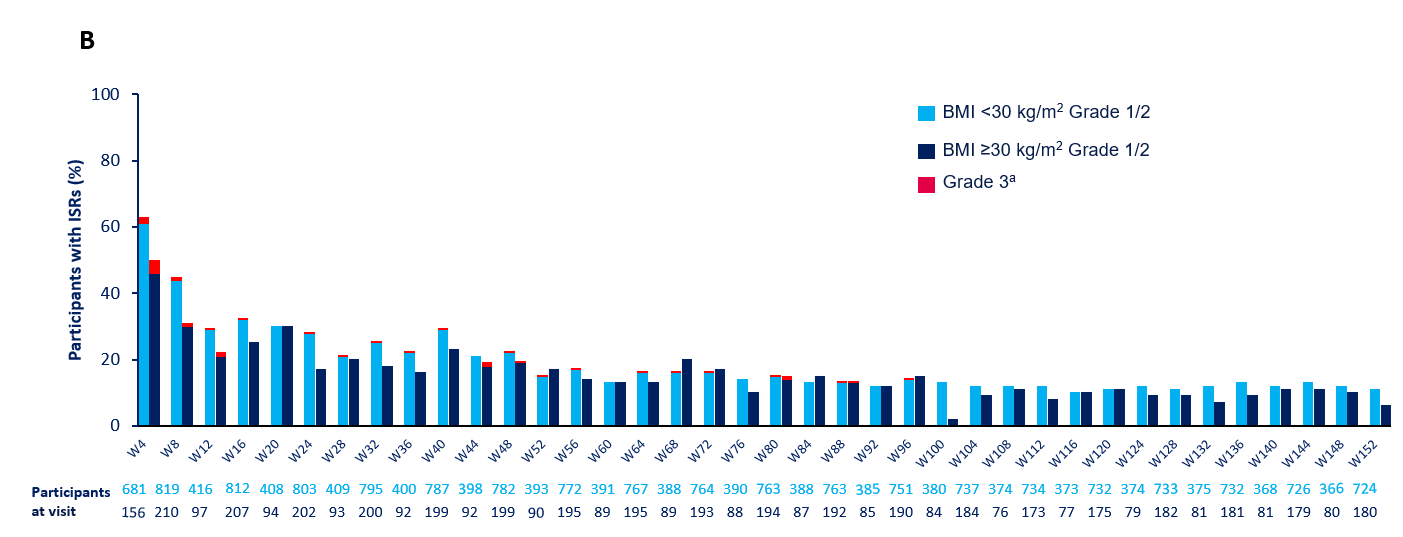


^a^AE grade is the maximum grade reported by the participant at each visit. Few ISRs were classified as Grade 3 (~1% of ISR events). There were no Grade 4 or 5 ISR events.

AE, adverse event; BMI, body mass index; ISR, injection site reaction; W, week.

Table S1. Summary of CVF Events Through Week 48^a^

| **Parameter, n** | **Pooled CAB + RPV LA participants across  FLAIR, ATLAS and ATLAS-2M** | |
| --- | --- | --- |
|  | **BMI <30 kg/m^2^ (n=1033)** | **BMI ≥30 kg/m^2^**  **(n=213)**^b^ |
| CVF through Week 48 | 6^c^ | 8^d^ |
| No other baseline factors | 3 | 0^e^ |
| At least one other baseline factor | 3 | 8 |
| RPV RAMs alone | 0 | 3 |
| HIV-1 subtype A6/A1 alone | 2 | 4 |
| Both | 1 | 1 |

^a^One participant had oral CAB + RPV dosing interrupted due to a false-positive pregnancy test and, upon reinitiation of oral therapy, had suspected virologic failure that was confirmed; this participant is not included in the table as they were excluded prior to receiving the first dose of CAB + RPV LA injection.
^b^Of the 213 participants with a BMI ≥30 kg/m^2^, 185 had data available for HIV-1 subtype and RPV RAMs; among the 28 participants who were missing data for one or both of the other baseline factors, none met the CVF criterion.
^c^Q8W, n=1; Q4W, n=5. The total n-value includes one participant who had a non-protocol CVF, who was identified retrospectively.

^d^Q8W, n=4; Q4W, n=4.
^e^BMI ≥30 kg/m^2^ was the only baseline factor.

BMI, body mass index; CAB, cabotegravir; CVF, confirmed virologic failure; LA, long-acting; Q4W, every 4 weeks; Q8W, every 8 weeks; RAM, resistance-associated mutation; RPV, rilpivirine.

Table S2. Event-Level ISR Summary Through Week 48

| **Parameter** | **Pooled CAB + RPV LA participants across FLAIR, ATLAS, and ATLAS-2M** | | | |
| --- | --- | --- | --- | --- |
|  | **BMI <30 kg/m^2^** | | **BMI ≥30 kg/m^2^** | |
|  | **Q8W  (n=268)** | **Q4W  (n=764)** | **Q8W  (n=59)** | **Q4W  (n=154)** |
| Participants who received an injection, n (%)^a^ | 262 (98) | 749 (98) | 59 (100) | 153 (99) |
| Number of injections, n | 4290 | 20,109 | 938 | 4032 |
| ISR events, n^b^ | 1495 | 5160 | 270 | 824 |
| Pain, n (% of injections) | 1266 (30) | 4356 (22) | 201 (21) | 644 (16) |
| Nodule, n (% of injections) | 60 (1) | 230 (1) | 6 (<1) | 63 (2) |
| Induration, n (% of injections) | 34 (<1) | 163 (<1) | 9 (<1) | 32 (<1) |
| Grade 3, n (% of ISR events)^c^ | 13 (<1) | 63 (1) | 16 (6) | 14 (2) |
| Median duration (IQR), days | 3 (2, 4) | 3 (2, 4) | 3 (2, 5) | 3 (2, 5) |
| Participant withdrawal due to injection-related reasons,  n (% of participants with injections)^d^ | 11 (4) | 23 (3) | 0 | 3 (2) |

^a^Represents the number of participants who received at least one injection.
^b^A single injection could result in more than one ISR.
^c^There were no Grade 4 or 5 ISR events.
^d^Includes participants who discontinued due to ISR AEs and who withdrew from the study citing injection intolerability.
AE, adverse event; BMI, body mass index; CAB, cabotegravir; IQR, interquartile range; ISR, injection site reaction; LA, long-acting; Q4W, every 4 weeks; Q8W, every 8 weeks; RPV, rilpivirine.

Table S3. Summary of Snapshot Outcomes at Week 96 and Week 152 (ITT-E Population)

| **Parameter, n (%)** | **BMI <30 kg/m^2^** | | | **BMI ≥30 kg/m^2^** | | |
| --- | --- | --- | --- | --- | --- | --- |
|  | **FLAIR Week 96**  **Q4W**  **(n=243)** | **ATLAS-2M Week 152 Q8W**  **(n=409)** | **ATLAS-2M Week 152 Q4W**  **(n=425)** | **FLAIR Week 96**  **Q4W**  **(n=40)** | **ATLAS-2M Week 152 Q8W**  **(n=113)** | **ATLAS-2M Week 152 Q4W**  **(n=98)** |
| **HIV-1 RNA <50 copies/mL** | 211 (86.8) | 357 (87.3) | 368 (86.6) | 34 (85.0) | 99 (87.6) | 81 (82.7) |
| **HIV-1 RNA ≥50 copies/mL** | 6 (2.5) | 8 (2.0) | 3 (0.7) | 3 (7.5) | 6 (5.3) | 2 (2.0) |
| Data in window not below threshold | 3 (1.2) | 0 | 0 | 0 | 1 (0.9) | 0 |
| Discontinued for lack of efficacy | 3 (1.2) | 7 (1.7) | 2 (0.5) | 3 (7.5) | 5 (4.4) | 2 (2.0) |
| Discontinued for other reasons while not below threshold | 0 | 1 (0.2) | 1 (0.2) | 0 | 0 | 0 |
| **No virologic data** | 26 (10.7) | 44 (10.8) | 54 (12.7) | 3 (7.5) | 8 (7.1) | 15 (15.3) |
| Discontinued due to AE or death | 12 (4.9) | 21 (5.1) | 20 (4.7) | 0 | 2 (1.8) | 4 (4.1) |
| Discontinued for other reasons | 13 (5.3) | 22 (5.4) | 33 (7.8) | 3 (7.5) | 6 (5.3) | 11 (11.2) |

AE, adverse event; BMI, body mass index; ITT-E, intention-to-treat exposed; Q4W, every 4 weeks; Q8W, every 8 weeks.

Table S4. Summary of CVF Events After Week 48 (FLAIR Week 96^a^ and ATLAS-2M Week 152; ITT-E Population)

| **Parameter, n** | **BMI <30 kg/m^2^** | | **BMI ≥30 kg/m^2^** | |
| --- | --- | --- | --- | --- |
|  | **FLAIR  Week 96**  **(n=243)** | **ATLAS-2M Week 152**  **(n=834)** | **FLAIR  Week 96**  **(n=40)** | **ATLAS-2M Week 152**  **(n=211)** |
| CVF total | 0 | 9 | 3 | 5 |
| CVFs after Week 48 | 0 | 3 | 0 | 0 |
| No other baseline factors | N/A | 1 | N/A | N/A |
| At least one other baseline factor | N/A | 2 | N/A | N/A |
| RPV RAMs alone | N/A | 1 | N/A | N/A |
| HIV-1 subtype A6/A1 alone | N/A | 1 | N/A | N/A |
| Both | N/A | 0 | N/A | N/A |

^a^One participant had oral CAB + RPV dosing interrupted due to a false-positive pregnancy test and, upon reinitiation of oral therapy, had suspected virologic failure that was confirmed; this participant is not included in the table as they were excluded prior to receiving the first dose of CAB + RPV LA injection.

BMI, body mass index; CVF, confirmed virologic failure; ITT-E, intention-to-treat exposed; N/A, not applicable; RAM, resistance‑associated mutation; RPV, rilpivirine.

Table S5. Safety Summary (Excluding ISRs) Through Week 96 and Week 152

|  | **BMI <30 kg/m^2^** | | | | **BMI ≥30 kg/m^2^** | | | |
| --- | --- | --- | --- | --- | --- | --- | --- | --- |
|  | **FLAIR^a^  Week 0–48**  **(n=243)** | **FLAIR^a^  Week 48–96 (additional participants since Week 48)^c^** | **ATLAS-2M^b^  Week** **0–48**  **(n=834)** | **ATLAS-2M^b^  Week 48–152 (additional participants since Week 48)^c^** | **FLAIR^a^  Week 0–48**  **(n=40)** | **FLAIR^a^  Week 48–96 (additional participants since Week 48)^c^** | **ATLAS-2M^b^  Week 0–48**  **(n=211)** | **ATLAS-2M^b^  Week 48–152 (additional participants since Week 48)^c^** |
| Any AE | 208 (86) | 20/35 (57) | 682 (82) | 86/152 (57) | 34 (85) | 2/6 (33) | 161 (77) | 29/50 (58) |
| Drug-related AEs | 66 (27) | 16/177 (9) | 198 (24) | 63/636 (10) | 12 (30) | 1/28 (4) | 36 (17) | 12/175 (7) |
| Any Grade ≥3 AE | 19 (8) | 8/224 (4) | 44 (5) | 57/790 (7) | 0 | 2/40 (5) | 15 (7) | 12/196 (6) |
| Drug-related AE | 4 (2) | 0/239 (0) | 9 (1) | 7/825 (1) | 0 | 0/40 (0) | 0 | 4/211 (2) |
| AE leading to withdrawal | 8 (3) | 4/235 (2) | 17 (2) | 14/817 (2) | 0 | 0/40 (0) | 1 (<1) | 5/210 (2) |
| Any serious AE | 11 (5) | 11/232 (5) | 33 (4) | 37/801 (5) | 0 | 2/40 (5) | 12 (6) | 10/199 (5) |
| Drug related | 1 (<1) | 0/242 (0) | 3 (<1) | 3/831 (<1) | 0 | 0/40 (0) | 0 | 0/211 (0) |

^a^FLAIR participants received CAB + RPV LA dosed Q4W.  ^b^ATLAS-2M participants received CAB + RPV LA dosed Q8W or Q4W.
^c^Participants having events with onset during FLAIR Week 48–96 and ATLAS-2M Week 48–152, with no events in the Week 0–48 period. Percentages are derived from the number of participants with no events in the Week 0–48 period.
AE, adverse event; BMI, body mass index; CAB, cabotegravir; ISR, injection site reaction; LA, long-acting; Q4W, every 4 weeks; Q8W, every 8 weeks; RPV, rilpivirine.
